# A Genome-Wide siRNA Screen in Mammalian Cells for Regulators of S6 Phosphorylation

**DOI:** 10.1371/journal.pone.0116096

**Published:** 2015-03-19

**Authors:** Angela Papageorgiou, Joseph Rapley, Jill P. Mesirov, Pablo Tamayo, Joseph Avruch

**Affiliations:** 1 Department of Molecular Biology, Massachusetts General Hospital, Boston, MA, 02114, United States of America; 2 Diabetes Unit, Medical Services, Massachusetts General Hospital, Boston, MA, 02114, United States of America, 617–726–6909; 3 Department of Medicine, Harvard Medical School, Boston, MA, 02115, United States of America; 4 Broad Institute of MIT and Harvard, 7 Cambridge Center, Cambridge, Massachusetts, 02142, United States of America; INSERM, FRANCE

## Abstract

mTOR complex1, the major regulator of mRNA translation in all eukaryotic cells, is strongly activated in most cancers. We performed a genome-wide RNAi screen in a human cancer cell line, seeking genes that regulate S6 phosphorylation, readout of mTORC1 activity. Applying a stringent selection, we retrieved nearly 600 genes wherein at least two RNAis gave significant reduction in S6-P. This cohort contains known regulators of mTOR complex 1 and is significantly enriched in genes whose depletion affects the proliferation/viability of the large set of cancer cell lines in the Achilles database in a manner paralleling that caused by mTOR depletion. We next examined the effect of RNAi pools directed at 534 of these gene products on S6-P in TSC1 null mouse embryo fibroblasts. 76 RNAis reduced S6 phosphorylation significantly in 2 or 3 replicates. Surprisingly, among this cohort of genes the only elements previously associated with the maintenance of mTORC1 activity are two subunits of the vacuolar ATPase and the CUL4 subunit DDB1. RNAi against a second set of 84 targets reduced S6-P in only one of three replicates. However, an indication that this group also bears attention is the presence of rpS6KB1 itself, Rac1 and MAP4K3, a protein kinase that supports amino acid signaling to rpS6KB1. The finding that S6 phosphorylation requires a previously unidentified, functionally diverse cohort of genes that participate in fundamental cellular processes such as mRNA translation, RNA processing, DNA repair and metabolism suggests the operation of feedback pathways in the regulation of mTORC1 operating through novel mechanisms.

## Introduction

The Target of Rapamycin (TOR) is a giant protein kinase that functions in two physically distinct, independently regulated complexes. TOR complex 1 (TORC1), the complex directly inhibited by rapamycin (when bound to FKBP12), is composed of TOR, raptor and mLst8, although the latter is apparently dispensable [1–3]. TORC1 is the major regulator of mRNA translation in all eukaryotic cells, and also controls autophagy, and transcription by all three RNA polymerases [4]. The regulation of TORC1 activity has been extensively studied; it is rapidly increased by numerous mitogenic and growth-promoting stimuli and by cell surface receptors and decreased by stressors of all types [4,5]. Many of these inputs operate by modifying the activity of the Tuberous Sclerosis complex (TSC), a GTPase activator for and negative regulator of Rheb [6]. Rheb is a ras-like GTPase that binds directly to and activates TOR in mammalian complex 1 [7]. Some inputs, most notably amino acid sufficiency, regulate mTOR predominantly downstream of the TSC [8]. We sought to apply genome-wide RNAi technology to examine whether novel mechanisms of TORC1 regulation in mammalian cells remained to be described. As a readout of mTORC1 activity, we used the phosphorylation of the 40S ribosomal protein, S6 [9].

S6 phosphorylation in mammalian cells is catalyzed by the protein kinase RPS6KB1, usually called p70 S6 [10,11], which in turn is activated by sequential phosphorylation by mTOR complex1 (most importantly at S6K1(Thr389) and PDPK1 [12,13]. Rapamycin, at low nanomolar concentrations, strongly inhibits S6KB1(Thr389) phosphorylation, kinase activity and S6 phosphorylation in essentially all cells examined in a highly selective manner [14]. S6K activity is especially sensitive to decreases in mTORC1 kinase activity, as shown by the ability of mTOR ATP site inhibitors to inactivate S6KB1(Thr389)at concentrations well below those needed to cause significant dephosphorylation of 4E-BP(Thr37/Thr46), another direct mTORC1 substrate [15]. Whereas the very low abundance of the S6K polypeptide renders S6KB1(Thr389) phosphorylation unsuitable for high throughput immunofluorescence assays of mTORC1 activity, 40S ribosomal subunits are highly abundant and rpS6 is the most specific and best characterized substrate of S6KB1. We used immunofluorescent imaging of Ribosomal protein S6 (rpS6) phosphorylation to screen for regulators of mTOR complex 1. S6 contains up to five sites of serine phosphorylation located near the polypeptide carboxyterminus that are phosphorylated by increasing S6KB1 activity in a sequential manner, in the order Ser236, 235, 240 244, 247 [9]. We employed a rabbit monoclonal antibody specific for RPS6(Ser235P/Ser236P) and high content microscopy to quantify rpS6 phosphorylation in the pancreatic cancer cell line MIA PaCa-2. Applying a stringent selection, we retrieved nearly 600 genes wherein at least two RNAi gave significant reduction in S6 phosphorylation. To identify mTORC1 regulators that did not require the TSC, we examined which of the elements identified in MIA PaCa-2 cells were also required for S6 phosphorylation in TSC1 null mouse embryo fibroblasts [16]. Among the 534 such gene products examined in TSC1 null MEFs, RNAi pools directed against 76 mRNAs were found to reduce S6 phosphorylation significantly in 2 or 3 replicates. Surprisingly, the only elements among this cohort previously associated with the maintenance of mTORC1 activity are two of the numerous subunits of the vacuolar ATPase subunits [17] and DDB1, which functions as a subunit of the CUL4 E3 ligase [18]. RNAis against another 84 TSC1null MEF targets were observed to reduce S6 phosphorylation in only one of three replicates. However, an indication that this group also bears attention is the presence of RPS6KB1 itself, as well as MAP4K3, a protein kinase that supports amino acid signaling of mTORC1 to RPS6KB1 [19] and Rac1 [20]. The retrieval in this screen of many gene products not previously associated with altered S6 phosphorylation indicates that mTORC1 is responsive to inputs from numerous intracellular sources through mechanisms that remain to be elucidated.

## Materials and Methods

### Chemicals and Reagents

General reagents were purchased from Sigma and Fisher while specific reagents were purchased from Sigma (Cycloheximide), Abcam (Thapsigargin), Calbiochem (Lactimidomycin) and Perkin Elmer (Protein labeling mix ^35^S).

### Cell lines and Culture conditions

MIA PaCa-2 cells (obtained from American Tissue Culture Collection) were cultured in Dubelcco’s Modified Eagle’s Medium (DMEM) (Gibco/BRL, Bethesda, MD) supplemented with 10% fetal bovine serum (FBS), Hyclone (Hyclone Laboratories, Logan, Utah), horse serum (Gibco/BRL) and 1X Penicillin/ Streptomycin. In experiments employing U2OS and Hela cells, cell culture, RNA and DNA transfection, cell extraction, immunoblotting and immunoblot quantification were carried out as described previously [15]. De novo protein synthesis was measured by the incorporation of medium ^35^S(Methionine+Cysteine) into TCA precipitable protein during 2 hours incubation.

### Antibodies

For siRNA screening of S6-P by immunofluorescence, S6 Ribosomal protein (Ser 235-P/236-P) rabbit monoclonal Antibody (2F9) (gift from Cell Signaling Technology), the Alexa Fluor 488 goat anti-rabbit IgG (H+L) highly cross-adsorbed (Invitrogen, Carlsbad, CA) were employed. For Western Blotting antibodies were purchased from Cell Signaling (anti-mTOR, anti-S6K-Thr^389^, anti-eIF2α-Ser^51^, anti-eIF2α, anti-4EBP-Ser^65^, anti-4EBP, anti-TSC1 and anti-TSC2), Immuno-Biological Laboratories (anti-raptor and anti-PRAS40), Abcam (anti-QARS and anti-Rheb), Proteintech (anti-LARS and anti-REDD1), Santa Cruz Biotechnologies (anti-S6K) and Sigma (anti-α-Tubulin).

### siRNA screening

A detailed description of the methods used in the primary screen has been published [21]. For the primary screen, we used the whole si-genome SMARTpool RNAi library Dharmacon library consisted of 21,121 siRNA SMART pools which represent known and predicted human genes and the majority of the human genome (Dharmacon siRNA library (Human genome, G-005000–05), Thermo Fisher Scientific, Lafayette, CO). Each RNAi pool consists of four individual oligonucleotides which target a different region of the same gene.

The Dharmacon library was screened in triplicate by transient reverse transfection of siRNAs into the Mia-Paca 2 cells. Each assay plate included non-specific targeting control RNAi, the FRAP and PLK1 siGENOME SMART pools. 4l of 1M RNAis were transferred from the library stock plates to each well of 384-well assay plates by using the Velocity 11 Bravo automated liquid handling platform at a final 100nM RNAi concentration. Lipofectamine 2000(Invitrogen) was used as the transfection reagent and diluted in Opti-MEM at a 1:100 ratio. Detailed description of transfection procedures employed are found in [21]. On each plate, a minimum of six positive (TOR) and six negative controls (NS) were placed. Cells were incubated for 72hr at 37C and subjected to IF protocol 72hr post transfection.

#### Immunofluorescence

Cells were initially washed with PBS (Gibco BRL, Cat No 11490) and then fixed in 4% paraformaldehyde at room temperature for 15minutes, washed 3X with PBS and subsequently permeabilized with methanol at-20C for 10 mins. Fixed cells were then blocked with a solution composed of 5% HS and 1% GS in PBS for 1 hour at room temperature. The primary PS6 antibody (0.32g) in blocking solution was then added and incubated overnight for 16hours at 4C using a shaking platform at low speed. The Alexa Fluor 488 goat anti-rabbit secondary antibody (Alexa 488; 1:1000 dilution of stock) diluted in block solution at a final concentration of 0.01mg/ml was then added. Cells were counterstained with DAPI and then washed three times with PBS.

#### Imaging Acquisition and Image Quantification

**Fig 1:**
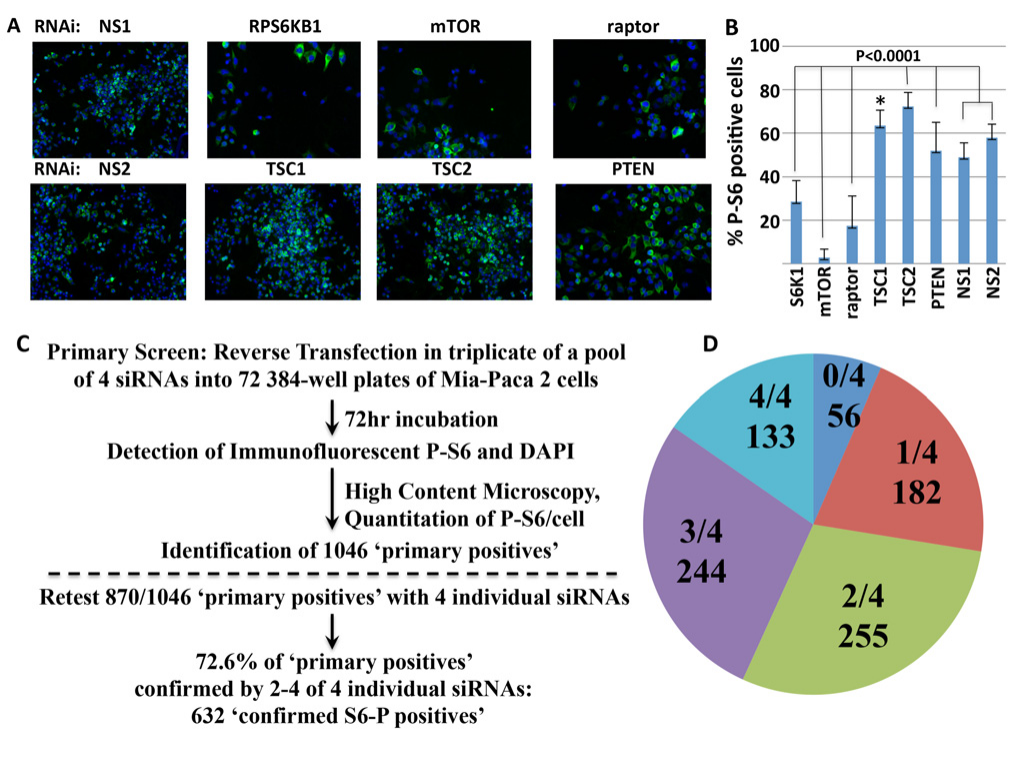
High-throughput image-based screens for genes regulating the phosphorylation of rpS6. A. Immunofluorescence analysis (IF) of rpS6Ser(235/236) phosphorylation. Mia-Paca 2 cells were transfected in 384-well plates with a control, nonspecific RNAis NS1 (upper panel) and (NS2) (lower panel), and RNAi pools directed at S6K, TOR and Raptor, TSC1, TSC2 and PTEN. After 72 hours they were fixed, permeabilized and stained by using a rabbit monoclonal anti-S6-P(Ser 235/236) primary antibody, detected with secondary anti-rabbit Alexa 488 antibody (green). Nuclei are stained with DAPI (blue). Representative images are shown. B. Quantitation of cytoplasmic S6-P levels. The bars indicate the % of total MIA PaCa-2 cells (estimated by nuclear count) that exhibit cytoplasmic S6-P immunofluorescence at an intensity above an arbitrary threshold (% S6-P positive cells; see methods). The z’ is 0.31 for the combined use of NS1 and NS2; because NS2 gave consistently higher z’ than NS1 (e.g., 0.45 vs 0.32 for the experiment shown) NS2 was used exclusively in the primary screen; error bars represent 1S.D. * = p<0.01. C. Flow chart of the primary screen: Summary of the screening, hit analysis and hit selection. 21,121 genes were tested using RNAis composed of pools of 4 RNAi oligos (Dharmacon Library); 72 384-well plates were screened in triplicate (See Table 1). The criteria for a “primary positive” are described in the text (Table 2 lists genes not scored due to severe inhibition of proliferation). D. Results of the confirmation screen. From the 1046 “primary positives”, 870 genes, including all 161 positive kinases and the top 709 ranked by Q (S3 Table), were examined in a confirmation screen wherein each of the four RNAis was tested individually. The pie chart indicates how many of the potential positive hits were confirmed by 0–4 individual siRNAs (listed in S4 Table).

Fixed, permeabilized cells were imaged on the High Content automated Imaging microscope ImageXpress Micro System (IXM) (Molecular Devices, http://www.moleculardevices.com) at 10X magnification (Fig. 1) and analyzed using the MetaXpress software program (Molecular Devices Inc). The Cell Scoring module was used for analysis and the primary readout for identifying hits was the percent positive cells. This assay focused on cytoplasmic staining. (For additional details see ref. 21). Images were analyzed and quantified by using the MetaXpress imaging software.

**Table 1:** Genes in Tsc1 null MEFs scoring positive in 2 or more replicates.

Gene Symbol	GeneID	Functional Class
Tagln3	56370	cytoskeleton-actin binding
Myl7	17898	cytoskeleton-myosin light chain
Myo3b	329421	cytoskeleton-non-muscle myosin
Tekt2	24084	cytoskeleton-spindle assembly
Tuba3	22144	cytoskeleton-tubulin
Dncic1	13426	cytroskeleton-Dynein subunit
Actl6b	83766	DNA modif-transcription-chromatin remodeling
Fen1	14156	DNA modification-flap structure-specific endonuclease 1
Smarcal1	54380	DNA modification-SWI/SNF related matrix associated, actin dependent chromatin regulator
Dnttip1	76233	DNA modification-terminal transferase interacting protein
Top3b	21976	DNA modification-topoisomerase 3
Wrn	22427	DNA modifocation-helicase
Shfm1	20422	DNA repair via BRCA2; also protein modification and RNA export
Ddb1	13194	DNA repair; protein ubiquitination
Mcm5	17218	DNA replication
Gpr1	241070	GPCR-chemerin
Ccr3	12771	GPCR-chemokine receptor
Gpr23	78134	GPCR-Lysophosphatidic acid
Olfr410	258702	GPCR-olf
Oxtr	18430	GPCR-oxytocin
Ffar3	233080	GPCR-short chain fatty acids
Gpr3	14748	GPCR-sphingosine 1-phosphate
Sphk1	20698	intracellualr signaling-sphingosine-1 kinase
Strada	72149	intracellular signaling- AMPK cofactor; STE20-related kinase adapter protein alpha
Pla2g2e	26970	intracellular signaling- Phospholipase A variant
Limd2	67803	intracellular signaling-binds protein kinase ILK
Edaradd	171211	intracellular signaling-EDAR associated protein
Gnefr	27414	intracellular signaling-exocyst-secretion regulating guanine nucleotide exchange factor
Rgl2	19732	intracellular signaling-guanyl nucleotide exchanger for Ral GTPases
Pla2g3	237625	intracellular signaling-Phospholipase A variant
Bmp8a	12163	ligand-
Uvrag1	78610	membrane traffic-activates the Beclin1-PI(3)KC3 complex to promote autophagy
Rab3a	19339	membrane traffic-Rab GTPase
Tmem38b	52076	membrane transport—TRIC-B cation channel
Grik5	14809	membrane transport-ionotropic glutamate receptor
Atp6v1c1	66335	membrane transport-v-ATPase subunit
Atp6v1h	108664	membrane transport-v-ATPase subunit
Mfsd10	68294	membrane-transport-major facilitator superfamily domain-containing protein 10
Cel	12613	metabolism-carboxyl ester(lipid) esterase
Fabp4	11770	metabolism-fatty acid binding
Ugcg	22234	metabolism-UDP-glucose ceramide glucosyltransferase
Slc25a24	229731	mitochondria-calcium dependent ATP-Mg2 uptake to buffer mito calcium
Gtpbp8	66067	mitochondria-GTP-binding protein 8, Gtpbp8
Mut	17850	mitochondria-methylmalonyl CoA mutase
Pcca	110821	mitochondria-propionyl CoA carboxylase, alpha
Pdhx	27402	mitochondria-pyruvate dehydrogenase complex, component X
Uqcr10	66152	mitochondria-ubiquinol-cytochrome c reductase, complex III subunit
Mtor	56717	protein kinase
Capn3	12335	protein modification-calpain 3
Casp1	12362	protein modification-caspase 1
Mmp11	17385	protein modification-matrix metalloproteinase
Mmp12	17381	protein modification-matrix metalloproteinase
Adamts1	11504	Protein modification-matrix metalloproteinase like
Serpinb5	20724	protein modification-serine (or cysteine) peptidase inhibitor, clade B, member 5
Ube2k	53323	protein modification-ubiquitin-conjugating enzyme
Tnpo2	212999	protein traffic-cytoplamic nuclear transport
Tmem115	56395	receptor
Tmem121	69195	receptor
Cd19	12478	receptor-B cell Ag coreceptor
Fcgr4	246256	receptor-FC
Ifitm1	68713	receptor-interferon induced transmembrane protein 1
Cd163	93671	receptor-scavenger family
Rps24	20088	RNA-40S ribsosomal subunit
Nob1	67619	RNA-ribosome assembly
Ints7	77065	RNA-snRNA biogenesis
Urm1	68205	RNA-tRNA sulfuration-ubiquitin related modifier 1 homolog
U2af1	108121	RNA-U2 small nuclear ribonucleoprotein auxiliary factor
Yipf2	74766	transcription
Zfp524	66056	transcription factor
Batf2	74481	transcription-ATF2-like
Ccnk	12454	Transcription-cyclin K-RNA polymerase CTD kinases
Rax	19434	transcription-homeobox protein
Trp53bp2	209456	transcription-proapoptotic tumor suppressor ASPP2
Thoc7	66231	transcription-RNA export
Ssu72	68991	transcription-RNA polymerase II subunit A C-terminal domain phosphatase
Lypd1	72585	unknown-? Tumor suppressor

**Table 2:** Genes in Tsc1 null MEFs scoring positive in one of three replicates.

Gene Symbol	GeneID	Functional class
Fkbp5	14229	chaperone
Fkbp6	94244	chaperone
Actg2	11468	cytoskeleton-actin
Gmfg	63986	cytoskeleton-cofilin family
Smc1a	24061	DNA structure
Mcm3	17215	DNA-replication
Gpr82	319200	GPCR-lower food intake in KO
Gpr147	237362	GPCR-neuropeptide FF
Avpr1B	26361	GPCR-AVP
Crhr1	12921	GPCR-CRH
Celsr2	53883	GPCR-flamingo type EC domain
Gcgr	14527	GPCR-glucagon
Gpr48	107515	GPCR-LGR4
Lhcgr	16867	GPCR-LH
Mtnr1b	244701	GPCR-Melatonin
Olfr15	18312	GPCR-olf
Olfr152	258640	GPCR-olf
Olfr42	18341	GPCR-olf
P2ry6	233571	GPCR-pyrimidinergic
Tact3	21338	GPCR-tachykinin
Vipr1	22354	GPCR-VIP
Fzd3	14365	GPCR-Wnt
Impa2	114663	intracellular signaling-inositol(myo)-1(or 4)-monophosphatase 2
Mapk8ip2	60597	intracellular signaling-kinase scaffold
Plcg1	18803	intracellular signaling-phospholipase C, gamma 1
Pde1b	18574	intracellular signaling- CM-dependent Pde1B
Orai2	269717	intracellular signaling-Ca^++^ release-activated Ca^++^ channel
Itpkc	233011	intracellular signaling-inositol-trisphosphate 3-kinase C
Ikbkb	16150	intracellular signaling-NFKB pathway
Seh1l	72124	intracellular signaling-NPC Nup107–160 complex; GATOR2 complex
Ptpdc1	218232	intracellular signaling-protein tyrosine phosphatase domain containing 1
Rac1	19353	intracellular signaling-small GTPase
Bcar3	29815	intracellular signaling; SH2 domain
Calb1	12307	intracellulat signaling-cytosolic Ca^++^ binder/buffer
Akap7	268287	intrecellular signaling-PKA anchor protein
Ccl4	20303	ligand-chemokine
Vwc2	319922	ligand-secreted BMP antagonist
Srpx2	68792	matrix-Chondroitin sulfate proteoglycan
Dspp	13517	matrix-dentin sialophosphoprotein
Cln3	12752	membrane structure-lysosomal
Nsmaf	18201	membrane structure-neutral sphingomyelinase activation associated factor
Pik4cb	107650	membrane structure-phosphatidylinositol 4-kinase, beta
Tjp2	21873	membrane structure-tight junction protein
Slc35f1	215085	membrane transport-?nucleotide sugars
Slc44a4	70129	membrane transport-thiamine pyrophosphate transporter.
Pfkfb3	170768	metabolism-6-phosphofructo-2-kinase/fructose-2,6-biphosphatase 3
Coasy	71743	metabolism-CoA synthase
Gpx2	14776	metabolism-glutathione peroxidase 2
Pank4	269614	metabolism-pantothenate kinase 4
Galntl4	233733	metabolism-polypeptide N-acetylgalactosaminyltransferase 18
Tpk1	29807	metabolism-thiamin pyrophosphokinase 1
Nfu1	56748	mitochondria-NFU1 iron-sulfur cluster scaffold homolog
Aldh18a1	56454	mitochondrial-proline, ornithine and arginine biosynthesis
Pink1	68943	mitochondrial-protein kinase
Ufd1l	22230	protein degradation-modification
Cdk3	69681	protein kinase-cell cycle regulation
Irak3	73914	protein kinase-IL1 receptor associated
Pim2	18715	protein kinase-overlaps specificity of Akt
Ripk5	213452	protein kinase-DUSTY like
Src	20779	protein kinase-tyrosine
Stk23	56504	protein kinase-SRSF proteins
Map4k3	225028	protein kinase-GLK
Map4k1	26411	protein kinase-HPK1
Map3k10	269881	protein kinase-MLK2
Rps6kb1	72508	protein kinase-S6
Cd3e	12501	receptor-T cell Ag
Acvr2	11480	receptor-ActivinA type
Fgfr3	14184	receptor-FGF
Cd244	18106	receptor-NK cell
Cd8a	12525	receptor-T cell
Rbm12	75710	RNA binding protein
Tdrd12	71981	RNA-piRNA biogenesis factor
Rpp25	102614	RNA-ribonuclease P subunit
Tnrc6	233833	RNA-RNAi and miRNA
Tars2	71807	RNA-Tars2 threonyl-tRNA synthetase 2, mitochondrial (putative)
Eif2b3	108067	RNA-translation initiation factor
Bhlhb9	70237	transcription factor
Nrbp	192292	transcription- nuclear receptor binding protein 1
Ccdc134	76457	Transcription-Cotranscriptional regulator-? Secreted protein
Tlx3	27140	transcription-homeobox protein
Med25	75613	Transcription-mediator of RNA polymerase II transcription, subunit 25
Smr3a	20599	submaxillary gland androgen regulated protein 3A
Sbsn	282619	unknown
Trabd	67976	unknown-TraB domain containing, TRABD

#### Data Analysis and Hit Scoring

Analysis of RNAi screening data was performed by use of the spotfire software program licensed through TIBCO Software Inc. http://www.tibco.com webcite; Spotfire DecisionSite 9.1.1 for Lead Discovery (TIBCO, Palo Alto, CA).

To identify hits, we used a level of “% positive cells” six standard deviations above or below that of the average value of “% positive cells” in all wells on the plate transfected with a scrambled RNAi, minimally 16 wells/plate. The z score was calculated by using the formula:

z= (X-AVG)/(y*Std dev)

where X = “% positive cells” value corresponding to the specific well, AVG = average value obtained from all wells on the plate transfected with a scrambled RNAi, y = the number of Std. Dev chosen, i.e., 6, * means “times”.

**Fig 2:**
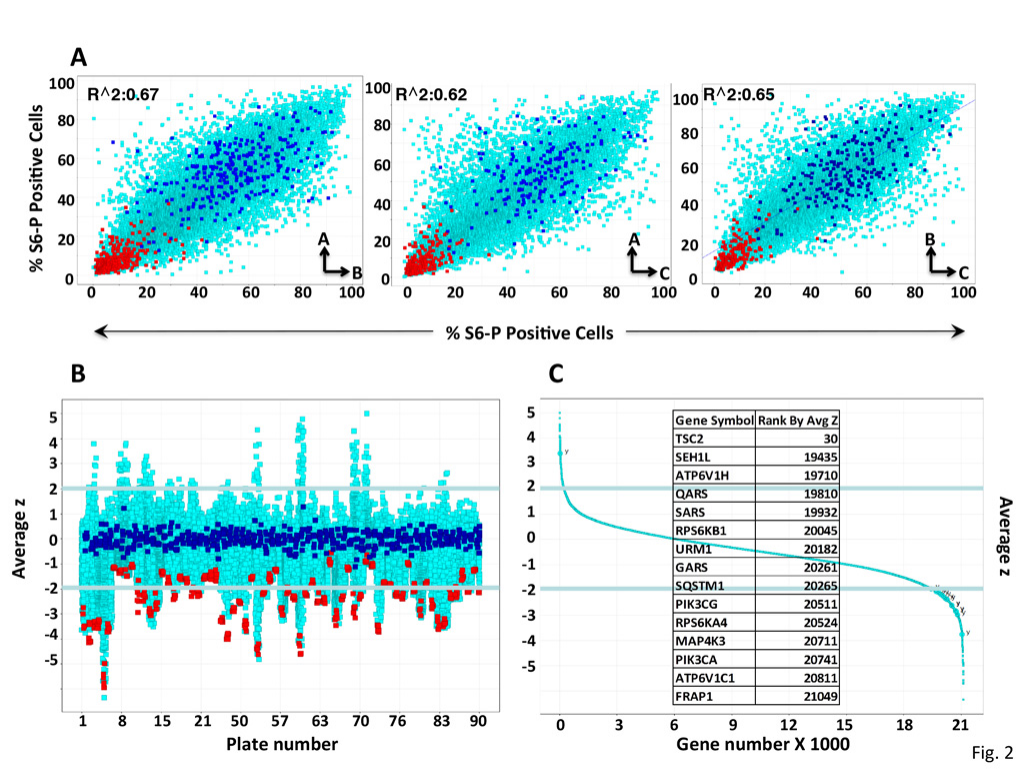
Indicators of Performance in the Primary Screen. A. Scatter plots comparing the percent S6-P positive cells in replicate plates for the entire screen. For each gene, the % S6-P positive cells in one plate is plotted against the value observed in a replicate plate (AvsB, AvsC, BvsC). Blue and red squares are values of %S6-P positive cells after transfection of scramble and mTOR siRNAs respectively, whereas aqua squares correspond to all other genes. The correlation coefficient (R^2 value) range from ~0.62 to ~0.67. B. Distribution of averaged z scores for all genes across the entire screen. Scatter plot comparing the z scores (y axis), averaged for all three replicates plates (numbered on the x-axis) for all genes across the entire screen. Color coding as in A. The cutoff of z+/- 2 is highlighted. Individula plates whose z’ was greatly inferior to replicates were eliminated from scoring (see Text and methods). C. Rank order plot. The averaged z-score for all replicates of all genes screened; primary positives were considered those exhibiting a z score exceeding +/- 2 in 2 or more replicates (~75% of genes) or on one plate chosen because either it was the only plate recovered or it exhibited a z’ of >0.4 over the replicates (~25% of genes). The position of selected genes is shown in the rectangular box.

We selected “potential positives” (S1 Table) employing the following three methods of analysis: i)-Hits obtained using the results from all three replicate plates where the absolute value of the z score for the corresponding candidate was equal or above 2 using 6S.D. on at least 2 plates;(37 Plates). ii)-Hits obtained using the results of 2 plates where the absolute value of the z score for the corresponding candidate was equal or above 2 using 6S.D.-this includes plates where a third replicate was lost entirely or was technically unsatisfactory (z’ < -0.05); (15 Plates). iii)-Hits obtained by analysis of one plate-because two replicates were lost or one replicate was lost and the second technically unsatisfactory, or because one replicate showed a z’ that was far superior in technical quality, with a Z’ >0.4 higher than either that of the two other replicate(s); (16 Plates). The potential positive hits were visually confirmed to exclude artifacts that may have skewed the percent positive PS6 values. Wells with out-of-focus images- obvious by visual inspection and as evident by discordance in the average total cell number among the three plates were also excluded. Genes for which the cell number was decreased by a minimum of two standard deviations corresponding to mTOR, were excluded as potential positives so as to eliminate proliferative failure for reasons other than inhibition of mTOR (S2 Table). Although the false negative rate cannot be estimated with accuracy, it is likely the stringent selection led to rejection of true regulators of S6 phosphorylation. In addition, the z values for the mTOR RNAi positive control averaged across the three replicate plates are more dispersed than the averaged scramble controls and ~38% are less negative than the chosen z cutoff of-2, i.e., scores that would be designated “negative” (Fig. 2).

For all of the wells for which the z was at or above the cut-off value (z = +/- 2), we calculate the probability corresponding to each z by using the formula

Q=(1-x)*100

where x = Absolute value (1-(1-NormalDistribution(z))*2). Subsequently, the cumulative probability considering all three assay plates that corresponds to a specific hit was obtained by taking the average of all probabilities.

#### Confirmation of potential positives in the primary screen

For the confirmation phase, 870 of the 1046 potential positives were retested in the confirmation phase of the primary screen (S3 Table) using the four deconvoluted siRNAs from each SMART pool separately at 100nM concentration. A similar protocol was followed as in the primary screen and the potential positives were screened in triplicate. Each plate included a minimum of 12 wells of the NS, TOR and 2 wells of PLK1. The rate of confirmation by 2 or more individual RNAis was very similar for 75

% of “potential positives” identified by concordance of two or more plates as compared with the 25% of “potential positives” identified based on one plate. As regards the false positive rate in the initial screen, the finding that 56 of the 870 “primary positives” failed to be confirmed by any of the individual RNAis from the initial RNAi pool provides a minimal estimate of 6.4%. The 182 “primary positives” confirmed by only 1 of 4 individual RNAis from the initial pool comprise a mixture of false and true positives in unknown proportion; assuming all to be false positive, the maximal estimate of false positive rate is 27.4% (S4 Table).

#### Secondary screen

The protocol for RNAi screening in the TSC1 null MEFs was similar to that employed in the primary screen except that RNAi pools were 75nM final concentration. Also, due to the greater variance observed for the positive and negative controls, the criterion employed for positivity, i.e., a “% S6-P positive cells” two standard deviations from the mean value of the corresponding NS value, is less stringent than that used for the MIA PaCa-2 cells.

#### RT-PCR

Total RNA was prepared using the RNeasy mini kit (QIAGEN). RNA (ug) was used for cDNA synthesis using SuperScript First-Strand Synthesis System for RT-PCR (invitrogen).

### Bioinformatic analysis

**Fig 3:**
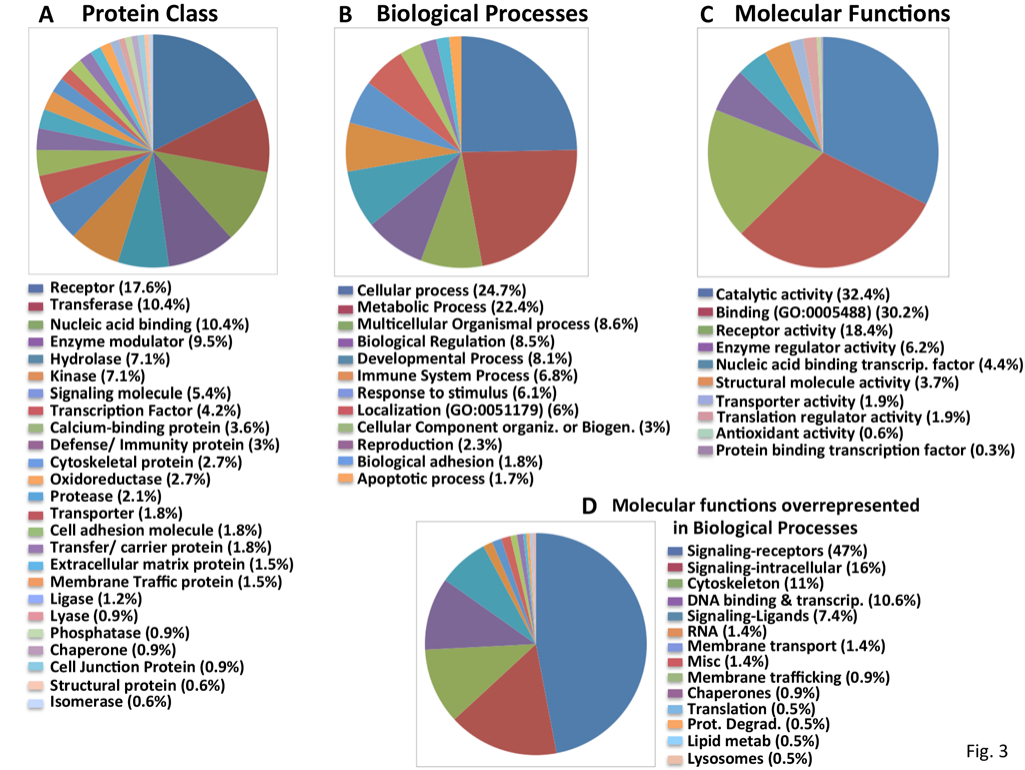
Classification of the “Confirmed S6-P positives” into functional groups. Categorization of “Confirmed S6-P positives” using the PANTHER classification system into A. Protein Class; B. Molecular Function; C. Biological Processes. D. Manual reclassification by molecular function of the non-redundant “Confirmed S6-P positives” (listed in Supp Table 7) comprising the subcategories of “Biological Processes overrepresented in comparison to the whole genome (shown in S1 Fig.).”

The Panther Classification system was used to categorize hits into molecular function, Biological processes, Protein Class [22] using the functional classification tool. To determine which categories were enriched in the confirmed hit gene list relative to their representation in whole human genome, the statistical overrepresentation test tool was used and p values were computed using the hypergeometric probability distribution. To access the statistical enrichment or overrepresentation of these categories for the hit genes relative to their representation in the whole human genome and the global set of genes examined in the siRNA screen by using the overrepresentation tool in panther (Fig. 3). Categories with a p-value of <0.05 was considered significant. Additional databases and software programs were used to validate the results obtained by PANTHER such as: the Human Protein Reference Database (HPRD), the Ingenuity pathway database, the Biomolecular Interaction Network Database (BIND), the Genego software program.

## Results

### Primary screen- MIA PaCa-2 human Pancreatic adenocarcinoma-derived cells

The MIA PaCa-2 cell line was chosen for the high throughput screen because of its constitutively high rpS6 phosphorylation levels [23], suitability for immunofluorescent imaging (Fig. 1A), adequate transfectability and predictable proliferative behavior. They are a hypotriploid human cell line whose modal chromosome number is 61. Sixteen to 20 marker chromosomes are commonly found in a cell. A few normal chromosomes are absent. These cells have a KRasG12C mutation and KRas depletion is proapoptotic. There is also loss of CDKN2A and a p53 (R248W) gain-of-function [24] oncogenic mutation that inhibits ATM catalyzed DNA repair [25].

#### RNAi Screening

21,121 genes were screened in triplicate using pools containing 4 RNAi oligonucleotides. Scramble RNAi against a non-specific targeting sequence served as the negative control and mTOR RNAi as the positive control [21]. Wells were scored by estimation of the % of cells (DAPI-stained nuclei) whose cytoplasmic rpS6-P fluorescence exceeded a value, optimized to yield the highest signal/noise ratio and optimal plate Z’. Wells containing scramble RNAi averaged 50–60% %S6-P positive cells, whereas those containing mTOR RNAi averaged 5–10% S6-P

positive cells (Fig. 1B). Experimental wells scored as positive were those showing a % S6-P positive cells that was higher or lower than the averaged % S6-P positive cells of the scramble wells on the same plate by a z equal or greater than +/-2, calculated using 6S.D. The screen was performed in triplicate and those genes scoring positive on two or three plates were considered “potential positives” and accounted for 75% of all “potential positives.” Approximately 25% of the “potential positives” were called on the basis of one plate, either because it was the only replicate remaining or because one plate was far superior in technical quality (z’ of >0.4 over the replicates), usually because the discounted replicates plates exhibited wide variance in the values for the scramble RNAi (S1 Table).

To exclude the possibility that knockdown of the putative TOR regulators causes toxicity, and to correct for decreased viability effects induced by RNAis we normalized for total cell number as measured by DAPI. Hits for which the average total cell number was 2 S.D. below the average total cell number corresponding to the mTOR positive control were excluded and considered to cause mTOR-independent proliferative failure.

The 95 targets of these RNAi pools (S2 Table) included for e.g.,10 proteins of the 40S ribosomal subunit, several RNA splicing factors, proteasome subunits. These criteria yielded 1046 “potential positives” (Fig. 1C) of which 220 RNAis resulted in increased rpS6 phosphorylation. It is likely that the relatively high baseline rpS6 phosphorylation levels in the MIA PaCa-2 cells biases against identification of targets whose depletion results in increased S6 phosphorylation.

#### Confirmation of potential positives

All 1046 “potential positives” were ranked by the Q value which represents the probability of a hit occurring by chance considering all replicate values (S3 Table, first tab). Of these, 870 were chosen for retesting with each of the four individual RNAi oligonucleotides comprising the original pool; this included all 161 “potential positives” from the kinase collection and strongest 709 of the remaining “potential positives.” Consequently, 176, lower confidence “potential positives” were not further evaluated (S3 Table, second tab). In addition, potential positives among the genes identified only by LOC (~2177), FLJ (~450; http://flj.lifesciencedb.jp/top/) or ORF (~1120) designations (see S1 Table) were excluded from further analysis. Here again we used a criterion of altered % positive cells exceeding a z score of +/-2, which corresponds to 6S.D. the value observed for the scramble RNAi. Six hundred thirty two of 870 (72.6%) were confirmed in 2, 3 or all 4 out of 4 individual RNAi (Fig. 1D). These are henceforth considered as confirmed “S6-P positives” (S4 Table).

#### Reproducibility of replicate plates and data quality

Some features of the data derived from the primary screen are shown in Fig. 2. Comparison of the “% positive S6-P” scores observed between plates yielded R^2^ values of 0.67 (AvsB) and 0.65 (BvsC) and 0.61 (AvsC), an acceptable level of concordance (Fig. 2A). A plot of the z value for all of the replicate plates used for scoring the primary screen, as described above, shows that whereas the averaged z values of replicate plates for the scramble RNAis tend to cluster tightly around zero, the averaged z values for the mTOR RNAi positive control are much more dispersed (Fig. 2B). A plot of all 21,121 genes according to the average z value is shown is shown in Fig. 2C (and listed in S5 Table).

#### Confirmed and proposed regulators of PS6 & mTORC1

It is of interest to examine how confirmed and proposed regulators of S6 phosphorylation and/or mTORC1 performed in the primary RNAi screen. The raw values for %S6-P positive cells and corresponding z for selected candidates are shown in Table S6. Among the core elements, the RNAi pools for S6K1B, mTOR and TSC2 scored as “potential positives”; S6K1B and mTOR but not TSC1 were confirmed in 2 or more individual RNAis. The TSC1 RNAi pool produced an increase in %S6-P positive cells to nearly the same level as did TSC2, but failed to qualify as a “potential positive” due to an insufficient z. Although the RNAi pools directed at Rheb [7] and RhebL1 gave considerable reduction in %S6-P positive cells, as with TSC1 the low z scores (corresponding to Rheb and RhebL1 knockdown) prevented their designation as hits. As regards raptor/KIAA1303, two of the three replicates were lost and although this RNAi pool was effective, as shown in Fig. 1B no inhibition was induced by this RNAi pool in the single high throughput replicate. Although the RNAi pools for RagA and RagC [26,27] depressed the %S6-P positive cells, none of the z values for any of the Rag GTPases RNAis reached the cutoff. The RNAi pools directed at each of the five Ragulator/LAMTOR subunits [28,29] and that for VPS39/hVAM6 gave no decrease in the %S6-P positive cells. The GATOR 1 complex (nprl2/nprl3/DEPDC5) is reported to be a negative regulator of amino acid-stimulated, Rag dependent mTORC1 activation, whose inhibitory effect is relieved by the GATOR2 complex (Sec13/Mios/WDR24/ WDR59/SEH1L) [30]. RNAi toward GATOR1 components did not significantly increase %S6-P positive cells and paradoxically, DEPDC5 RNAi strongly reduced %S6-P positivity, although with a z far less than-2. Among the five components of GATOR2, only the RNAi pool directed at SEH1L gave significant inhibition of %S6-P positive cells, which was strongly confirmed with the individual RNAi. Other elements reported to regulate mTORC1 include putative positive regulators MAP4K3 [19], SQSTM1/p62 [31], V-ATPase [17], PAT1/SLC36A1 [32], folliculin/FNIP1 [33] and the negative regulator SH3-BP4 [34]. Among this latter cohort, the RNAi pools for MAP4K3, SQSTM1 and the v-ATPase subunits V1H and V1C were confirmed “S6-P positives.”

#### Bioinformatics

The 632 confirmed “S6-P positives” were analyzed using the PANTHER program [22] and their distribution according to Protein Class, Biologic Process and Molecular Function is displayed in Fig. 3. Twenty five Protein Classes are represented (Fig. 3A). The subcategories of GPCRs, transmembrane receptors, protein kinases/transferases and transporters are overrepresented among the “S6-P positives” as compared to the entire genome (S1 Fig.). Approximately 80% of the “S6-P positives” from the Biologic Processes (Fig. 3B) and Molecular Functions (Fig. 3C) fall into 3–6 broad categories. Further analysis of the subcategories comprising Molecular Functions identified the same functional subgroups found to be overrepresented in the Protein Class group (S1 Fig.). However, further analysis of the “S6-P positives” within “Biological Processes” identifies more numerous and seemingly diverse subcategories (S1 Fig.). Because these subcategories of Biologic Processes contain overlapping components, we identified the 217 nonredundant “S6-P positives” in those subcategories (S7 Table) and manually reclassified them by molecular function (Fig. 3D). Not surprisingly, GPCRs and other receptors again comprise nearly half of these elements followed by intracellular signaling proteins, especially protein kinases.

#### Confirmed “S6-P positives” are enriched in “mTOR essential” genes

**Fig 4:**
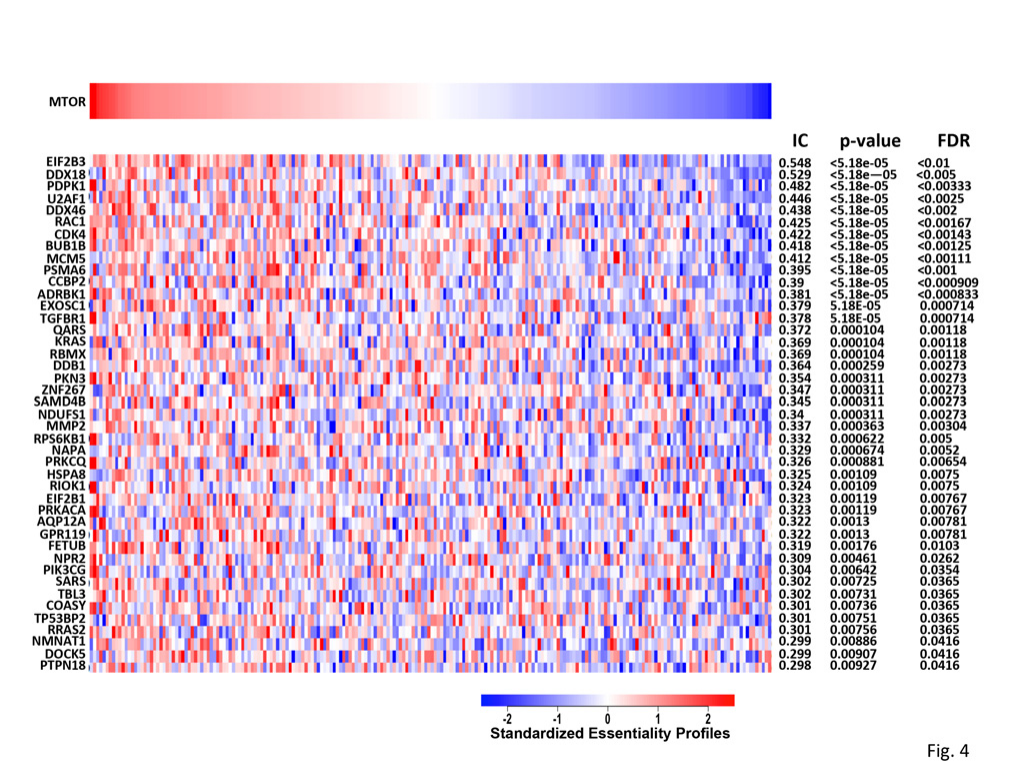
Confirmed S6-P positives whose depletion affects the viability of a panel of cell lines similar to that of mTOR depletion. This heat map shows the standardized essentiality profiles (blue more essential, red less essential) of 43 S6-P positives genes that are also significantly associated (FDR < 0.05) with mTOR essentiality (left). The association is determined by the normalized mutual information (IC score, nominal p value and FDR are shown at the top).

Seeking independent verification that the “confirmed S6-P positives” are enriched in regulators of mTORC1 we utilized the Achilles dataset [35; release 2.4.1 available at www.broadinstitute.org/achilles)] which enabled an inquiry of how shRNA mediated depletion of “confirmed S6-P positives” affects the proliferation/viability of a large number of cancer cell lines in comparison to depletion of mTOR. Among the 632 “confirmed S6-P positives,” 193 have been analyzed in 216 cell lines. For each gene in this subset of “confirmed S6-P positives” its essentiality in each of the 216 cell lines was determined [36,37] and matched against the essentiality profile for mTOR itself (Fig. 4) using as association statistic the normalized mutual information [38]; nominal p-values and False Discovery Rates (FDR) were computed using an empirical permutation test. From the 193 “confirmed S6-P positives,” 43 were found to match the mTOR essentiality profile in the 216 cancer cell lines at the 0.05 False Discovery Rate (FDR) level of significance. To assess whether the finding that 43 of 193 “confirmed S6-P positives” exhibit coessentiality with mTOR at this level of significance (FDR < 0.05, IC > 0.298) exceeds what would be expected from a random set of 193 genes, we applied a hypergeometric distribution test to the 6143 genes in the Achilles 2.4.1 dataset and obtained a p-value of 0.0025. Thus the randomly chosen subset of 193 “confirmed S6-P positives” is significantly enriched in genes whose depletion affects the viability of 216 cell lines in a manner paralleling that caused by mTOR depletion. Inasmuch as depletion of these 193 genes also suppresses S6 phosphorylation, we conclude that “confirmed S6-P positives” are significantly enriched in regulators of mTORC1.

In addition, the co-essentiality association revealed by this analysis indicates that cancer cell lines more sensitive to mTOR RNAi knockout are similarly sensitive to knockout of the 43 specific genes displayed in Fig. 4. These genes might help delineate a state characterized by co-dependency of mTOR and the respective S6-P positive genes in regards to cell proliferation and viability.

### Secondary screen-TSC1 null mouse embryo fibroblasts

#### Confirmation of S6-P positives in a secondary screen

**Fig 5:**
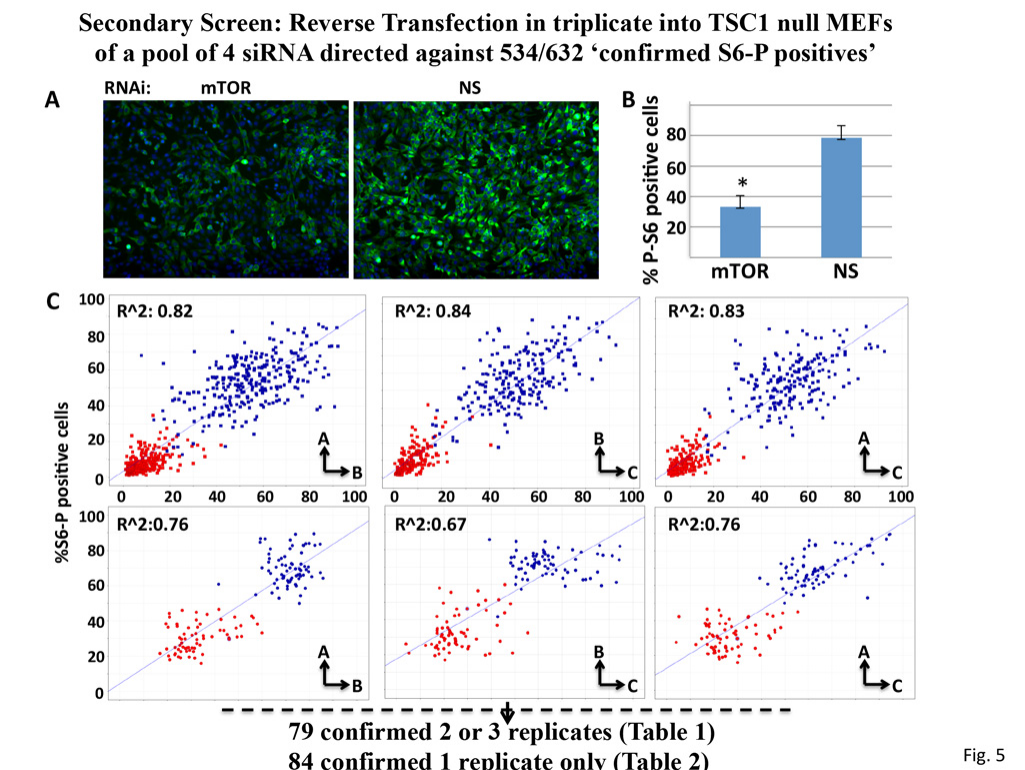
Performance Properties of the siRNA Screen using TSC1 null MEFs. A. Representative Immunofluorescence images of rpS6Ser (235/236) phosphorylation in TSC1 null MEFs transfected with nonspecific (NS) or mTOR-directed siRNAs. The cells were stained with DAPI (blue) and for rpS6(Ser 235P/236P) (green) as in Fig. 1A. B. Quantitation of “%S6-P positive cells” for the TSC1 null MEFs treated with nonspecific (NS) or mTOR-directed siRNAs. Values represent the average of 42 replicate wells +/- 1S.D, for each NS and mTOR siRNA; *p = 0.05 based on two-tailed student’s t-test. C. Comparison of MiaPaCa cells (upper three plots) and into TSC1 null MEFs (lower three plots) for “%S6-P positive cells” after transfection with nonspecific (blue squares) and mTOR (red squares) siRNA. Scatter plots comparing the positive (mTOR) and negative (NS) controls among replicate plates The MEFs exhibit higher average initial “%S6-P positive cells” but less potent and more variable suppression of “%S6-P positive cells” by mTOR siRNA.

In an effort to select among the large number of confirmed “S6-P positives” for further analysis, and to minimize the impact of technical factors, e.g., RNAi efficacy, protein target abundance and half-life, etc. we designed a secondary screen to determine which among the elements important for S6 phosphorylation in Mia PaCa-2 cells was important to the maintenance of phospho-S6 in a line of spontaneously immortalized TSC1 null mouse embryonic fibroblasts (MEFs) [16]. The high basal median S6-P positivity of TSC1 null MEFs (Fig. 5) interdicts evaluation of MIA PaCa-2 “S6-P positives” that increase S6-P, however this secondary assay, in addition to employing different RNAis in different cell backgrounds and requiring conservation of function across human and mouse, presumably eliminates stimulatory inputs acting through TSC and focuses attention toward pathways whose inputs are more likely to act between Rheb-GTP and rpS6-P.

Mouse RNAi pools corresponding to 534 (see S8 Table) of the 632 confirmed S6-P positives in the MIA PaCa-2 screen were available and were tested in triplicate. As compared with MIA PaCa-2 cells, the TSC1-null MEFs exhibit higher median “%S6-P positive” cells (Fig. 5A-C), but a more variable and less robust suppression of “%S6-P positive” cells in response to mTOR RNAi (Fig. 5C, compare the red squares in the upper panels to those in the lower panels). This likely reflects less efficient and more variable transfection and results in a smaller dynamic range of S6 phosphorylation. We therefore applied a less stringent selection criterion as compared with the MIA PaCa-2 cells, i.e. altered rpS6-P >2S.D. from the scramble on at least 2 out of the 3 replicate plates, which yielded 76 hits (Table 1); an additional 84 scored positive on 1/3 plates (Table 2).

Three of the mRNAs that showed significantly reduced %S6-P positive in 2 or 3 of 3 replicates (henceforth called MEF2/3 positives) (Shfm1, Urm1 and Atp6v1c1), in addition to Tor, are orthologous to genes identified by Parsons et.al. [39], whose deletion confers rapamycin sensitivity in haploid strains of S. cerevisiae. Both the MEF 2/3 positives (Table 1; U2af1, Mcm5, Ddb1 and Ptpn18) and the MEF single positives (Table 2; Eif2b3, Rac1, Rps6kb1, Coasy) include 4 genes that exhibit “co-essentiality” with mTOR in the Achilles database (Fig. 4).

#### Glutamine tRNA synthase/QARS

**Fig 6:**
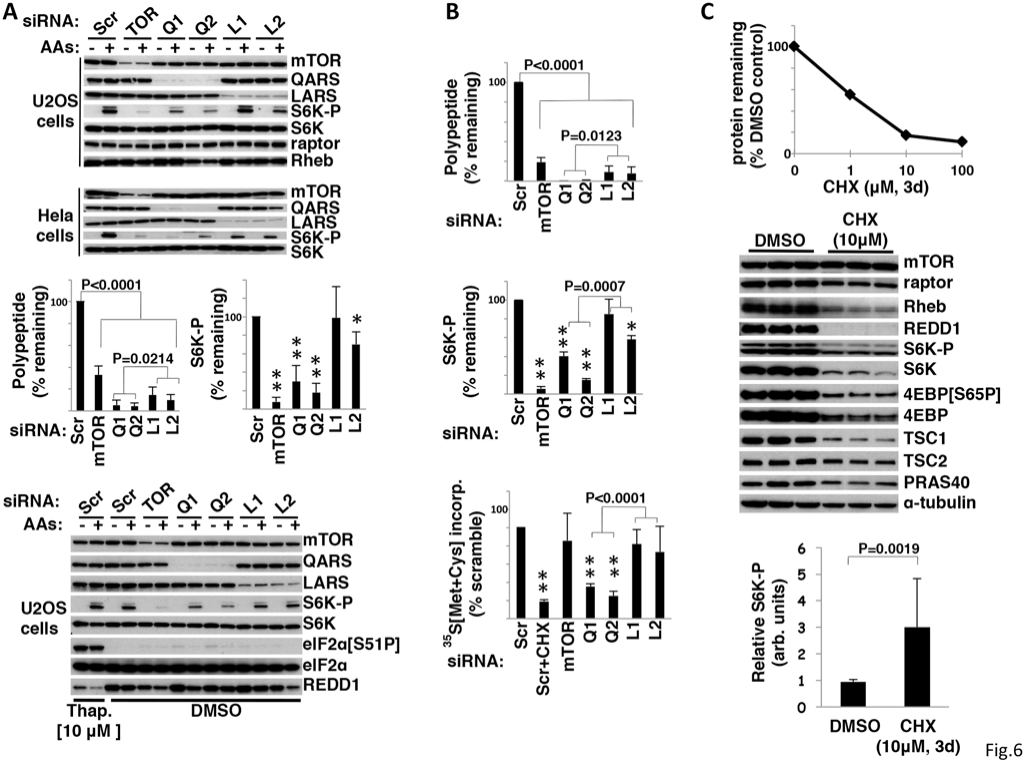
siRNA-mediated depletion of QARS inhibits mTORC1 signaling. A. The effect of mTOR, QARS and LARS siRNA upon polypeptide knockdown and S6K-Thr^389^ phosphorylation. U2OS and Hela cells were transfected with siRNA oligos against mTOR, QARS (Q1,Q2), LARS(L1,L2) and a scramble control. After 72h amino acids were withdrawn for 2 hours and added back for 15 minutes as indicated. Cells extracts were subjected to SDS-PAGE and membranes were immunoblotted with the antibodies as indicated. The bar graphs display the combined results of three experiments (mean-/+ 1 S.D.; ** = p<0.0001 and * = p<0.002 vs scramble) The experiment shown in the bottom panels compares the effects of Thapsigargin (10M) with the indicated siRNAs on S6K-P (fourth from top), eIF2α(Ser51-P) (third from bottom) and the abundance of REDD1 (bottom). B. siRNA-mediated depletion of QARS inhibits global protein synthesis. Graphical representation of the combined results from three experiments (mean-/+ 1 S.D.) examining the effect, relative to scramble siRNA, of siRNA against mTOR, QARS and LARS on the abundance of the target polypeptides (upper), the relative phosphorylation of S6K-P (middle; ** = p<0.0001 and * = p<0.002 vs scramble) and on overall protein synthesis (bottom) in nutrient and serum replete U2OS cells. Analyses were carried out three days after transfection. ^35^S[Methionine+cysteine] was added two hours before harvest; cycloheximide (CHX, 100μM) or carrier was added 30′ prior to ^35^S. C. The effect of inhibiting translation for three days on mTORC1 signaling. Graphical representation of a dose response of cycloheximide (CHX) on global protein synthesis in U2OS cells. U2OS cells were plated in DMEM with 10% FCS +/- CHX and fresh media containing carrier or CHX was added every 24 hours for 72 hours. The cell were harvested at 72 hours; protein content expressed as a fraction of carrier control is plotted in the upper graph. Immunoblots of cell extracts for the proteins indicated are shown in the middle and the ratio of S6K-P/S6K (mean-/+ 1 S.D.) is shown in the bar graph at the bottom.

We were intrigued by the finding that depletion of Glutamine tRNA synthase/QARS gives strong inhibition of S6-P in MIA PaCa-2 cells and is also high on the list of genes whose depletion affects the proliferation of the Achilles cancer cell cohort in a manner similar to mTOR depletion (Fig. 4). In contrast, RNAi directed at Leucyl tRNA synthase, which has been previously reported as a positive regulator of TORC1 [40], did not give significant inhibition of S6-P. Similar results were observed in U2OS and HeLa cells (Fig. 6A, top): in U2OS, 90–95% reduction of QARS reduced amino acid-stimulated S6K(T389) phosphorylation by 70–80%, whereas 85–90% depletion of LARS gave a 0–25% reduction (Fig. 6A, bar graphs). Despite the marked depletion of both QARS and LARS, the phosphorylation of eIF2α (Ser51) was not increased. The latter is an indication that the accumulation of uncharged tRNA, if any, was insufficient to activate the GCN2 kinase (Fig. 6A bottom). Depletion of either QARS or LARS did not alter levels of the Rheb, mTOR, raptor, or S6K polypeptides, nor was the abundance of the very short-lived polypeptide REDD1 diminished (Fig. 6A). These findings suggested that RNAi-induced depletion of these AARSs did not reduce protein synthesis. Although, this held true subsequent to LARS depletion, the same effect on protein synthesis was not observed for QARS depletion. Depletion of QARS polypeptide from serum and nutrient replete U2OS inhibited protein synthesis by 65–75% as measured by ^35^S-Met+^35^Cys incorporation into protein (Fig. 6B). QARS depletion led to inhibition of overall protein synthesis and S6K phosphorylation (T389-P), whereas comparable depletion of LARS inhibited neither. The differential effects on protein synthesis between the two AARS raised the question of whether reduced S6K phosphorylation (T389-P) caused by QARS depletion is due to the inhibition of protein synthesis. Brief treatment of cells with the inhibitor of global mRNA translation cycloheximide is known to activate mTORC1 [41], although the effect of sustained inhibition of protein synthesis of a magnitude comparable to that corresponding to QARS depletion is not known. U2OS cells incubated with cycloheximide (10μM for 3 days, to match the duration siRNA treatment) exhibit an 80–90% reduction in total protein accumulation as compared with cells incubated with carrier alone. Under these conditions, the relative abundance of S6K and Rheb are radically reduced, but phosphorylation of the mTORC1-catalyzed sites S6K (T389) is fractionally increased: a similar pattern is observed with lactidomycin, a selective inhibitor of translational initiation (not shown). Sustained but nonselective inhibition of protein synthesis does not inhibit and may activate mTORC1, an effect previously attributed to either an accumulation of cellular amino acids [41] or to loss of the TSC activator REDD1 [42]. It remains to be determined whether the inhibition of mTORC1 by QARS depletion reflects the loss of an mTORC1 activator(s) whose abundance is selectively sensitive to depletion of QARS (or Urm1) or to some non-translational action of QARS.

## Discussion

### Primary screen

The recovery of a subset of the known elements crucial for S6 phosphorylation together with the significant enrichment of the confirmed “S6-positives” among the genes whose essentiality parallels that of mTOR in a large cohort of cancer cell lines supports the view that the 642 genes identified in the primary screen for regulators of S6 phosphorylation are in fact enriched in regulators of mTOR complex 1. The most heavily enriched categories among the “S6-P positives” retrieved in the primary screen are the cell surface receptors, followed by intracellular signaling proteins, especially protein kinases. An enrichment of protein kinases among the “S6-P positives” is not surprising, inasmuch as multiple protein kinases are known to contribute to S6 phosphorylation, especially mTOR, RPS6KB1 and PDPK1 [13], which together with mTOR complex1 catalyzes RPS6KB1 phosphorylation and activation [12]. IKK [43] and PIM2 [44], known inhibitors of the TSC Rheb GAP activity, MAPK11/p38βwhich activates mTORC1 in response to arsenite by phosphorylation of raptor [45] and MAP4K3/Glk, which is implicated in amino acid regulation of mTORC1 [19] are among the S6-P positives as is Casein Kinase 2, which through its action on TEL2, is perhaps important for mTOR complex assembly [46]. Based on published literature, thin but plausible explanations can be inferred for several other of the protein kinases retrieved (e.g., PKC, PKC, PKA, BRSK2/SADA and six tyrosine kinases). Nevertheless, the mechanisms by which depletion of the many other protein kinases (ser/thr) diminishes S6 phoshorylation are not yet evident. The finding that S6 phosphorylation is influenced by an unexpectedly large number of protein kinases raises the possibility that a regulatory network far more complex than that currently envisioned operates upstream of RPS6K1B. Alternatively, despite the relatively high statistical stringency applied to hit selection, the possibility that many of these S6-P positives are in fact not regulators of S6 phosphorylation in the MIA PaCa-2 cells, i.e., are false positives, cannot be discarded.

The recovery of 83 GPCRs among the “S6-P positives” in the primary screen (e.g., Fig. 3D) was not anticipated. Also retrieved were the GPCR-specific intracellular signaling elements Transducin/GNAT2, Gsα/GNAS, GNB1,3 and 5, GNG3, 5 and 7 and beta arrestin-1/ARRB1. A GPCR regulated pathway to mTORC1 in MiaPaCa cells might operate through Gβγ activation of PI-3 kinaseγ/PI3KCG (an S6-P positive) or via β-arrestin recruitment of Src (also an S6-P positive) [47]. As there are nearly 800 GPCRs in the human genome, including nearly 400 olfactory receptors, some are likely to be retrieved as false positives in a primary screen. Nevertheless, the prevalence of GPCRs among the S6-P positives exceeds by several fold their representation in the human genome (~13% vs ~4%) and the basis for this enrichment is not known.

### Secondary screen

A relatively small number of perturbations have been shown thus far capable of inhibiting mTORC1 signaling in TSC null cells, including H2O2, sorbitol [48], energy stress (through AMPK phosphorylation of raptor [49] or PRAK phosphorylation of Rheb(Ser130) [50]) and withdrawal of ambient amino acids [51], through an incompletely understood mechanism that involves the RagA or B/C or D heterodimeric GTPases [26,27].

Focusing on the targets in the TSC1-null MEFs confirmed on 2 or more of 3 replicates (Table 1, S2A Fig.), this cohort has proportionately fewer GPCRs (9%) as compared with the confirmed “S6-P positives” from the MIA PaCa-2 cells (13%), and apart from mTOR no protein kinases; however, the 84 targets confirmed on only one of three replicates (Table 2) includes 14 GPCRs (so that GPCRs comprise 13% of combined MEF “S6-P positives,”) and 11 protein kinases, including S6KB1 and MAP4K3/Glk (S2B Fig.). The MEF2/3 cohort does include a diverse array of non-kinase signaling molecules that, with the exception of Stradα, have no known connection to mTORC1 signaling. Stradα is a pseudokinase scaffold necessary for the activity of Lkb1, an upstream activator of AMPK. Stradα depletion from mouse neural progenitor cells activates mTORC1 through loss of AMPK activity [52]. Inasmuch as AMPK inhibits mTORC1 both by activation of TSC [6] and by direct phosphorylation of raptor [49], it is unclear why depletion of Stradα inhibits S6 phosphorylation in TSC1null MEFs.

Among the MEF2/3 positives with a prior connection to mTORC1 are the v-ATPase subunits V1C1 and V1H, the ubiquitin-like polypeptide Urm1 and the WD40 domain protein DDB1; we have examined the relation of the vATPase subunits and URM1 with mTORC1 in additional experiments.

#### v-ATPase subunits V1H & V1C

The retrieval of v-ATPase subunits V1H and V1C as confirmed “S6-P positives” both in the MIA PaCa-2 cells and in the TSC1null MEFs is supportive of the role of the v-ATPase as a positive upstream mTORC1 regulator [17]. We confirmed the ability of RNAi to V1H (but not V1C1) to inhibit S6K(T389P) in U2OS cells (not shown). These v-ATPase subunits, which regulate rotation of the ATPase assembly, are on the cytoplasmic side of the lysosome and thus available to mTORC1. However we have been unable to detect a specific role for V1H and V1C subunits in mTORC1 localization or activation. In experiments not shown, we found that binding of recombinant V1H with coexpressed mTOR, raptor, Rheb or the Rag heterodimers is not detectable, whereas recombinant V1C1 proved generally sticky. Similarly overexpression of V1H and V1C1 does not modify the phosphorylation of co-expressed S6K in the presence or absence of amino acids, either when these v-ATPase subunits are expressed as the wild type polypeptides, or when directed to the relevant subcellular compartment either by carboxy terminal fusion of the Rheb carboxy terminal 15 amino acids, or amino terminal fusion of the p18/LAMTOR1 amino terminal 18 amino acids. Thus, the mechanism by which the v-ATPase promotes mTORC1 activity and the specific role of these subunits, if any, requires further study.

#### URM1

The retrieval of the ubiquitin-related modifier polypeptide Urm1 as a MEF 2/3 positive merited additional consideration both because of its unique dual function as both a protein and tRNA modifier and because its deficiency in S. cerevisiae has been shown repeatedly to confer sensitivity to rapamycin [39,53]. Activation of Urm1 occurs though the sequential action of NFS1, a cysteinyl sulfurase that thiolates a cysteine on the E1-like enzyme MOCS3. In turn MOCS3 cleaves the URM1 carboxterminal Gly-Gly in an ATP dependent manner, adenylating Urm1 and adding it covalently as a thiocarboxylate [54]. Urmylated MOCS3 can react with -lysine on proteins in response to oxidative stress [55] by a mechanism and specificity that are unknown, or via the mediation of the cytosolic thiouridylase CTU1-CTU2, thiolate uridine at position 34 (the wobble base of the anticodon) of the Glu, Gln and Lys tRNAs [54]. Notably, S. cerevisiae strains lacking Urm1, Uba4(MOCS3), Ncs6(CTU1) and Ncs2(CTU2) each exhibit rapamycin sensitivity [39]. Urm1 deficient strains can be rescued with the rapamycin-resistant TOR2(S1972I) mutant [56] in S. cerevisiae, or by overexpression of the Glu/Gln/Lys tRNAs, especially in combination in either budding [53] or fission [57] yeast. These findings point to an important role for mistranslation in the genesis of the rapamycin sensitivity.

In the primary and confirmation MIA PaCa-2 screens, both the RNAi pool against *URM1* and all of the four individual RNAi oligonucleotides led to strong inhibition of S6 phosphorylation. In subsequent experiments, URM1 knockdown by RNAi decreased S6K phosphorylation at Thr 389 in U2OS cells (not shown). The RNAi pools against *NFS1* and *CTU1* also gave strong inhibition of S6-P but with an insufficient z, whereas the RNAi pool against *MOCS*3 gave no inhibition and an RNAi pool against *CTU2* was not included in the screen (S1 Table). Because of these ambiguous results regarding the elements required for URM1 activation and U34 thiolation, we reexamined the impact of the original RNAi pools in comparison to *mTOR* RNAi. In eight experiments (data not shown) wherein mTOR RNAi inhibited S6-P in MIA PaCa-2 cells with z ≤ -2 each time, the RNAi pools for *NFS1*, *MOCS3*, *CTU1* and *CTU2* did so in 7/8, 5/8, 8/8 and 4/8 experiments, respectively. In the TSC1null MEFs the RNAi pool against *Urm1* gave strong inhibition of S6 phosphorylation, however this proved to be attributable to one of the four individual RNAis. We therefore examined an additional eight individual RNAis directed at *Urm1* in the TSC1null MEFs; altogether 3/12 RNAis gave consistent inhibition of S6 phosphorylation. Moreover, RNAi pools against *Mocs3*, *Ctu1* and *Ctu2* did not reduce S6-P in TSC1 null MEFs (data not shown). We concluded that the failure of U34 thiolation contributes to the inhibition of S6 phosphorylation in MIA PaCa-2 cells, presumably through mistranslation at one or more of the codons for Gln, Glu or Lys in one or more unknown proteins. In contrast to MIA PaCa-2 cells, URM1 deficiency does not impair mTORC1 signaling in TSC1 null MEFs.

#### DDB1

Interestingly, DDB1 is among the few mouse orthologs retrieved that overlaps with the dTORC1 regulators retrieved by Lindquist et.al. [58] from a genome wide dsRNA screen for S6-P regulators in Drosophila cells. Although first identified in the repair of UV-induced DNA damage, DDB1 is also a component of the CUL4 ubiquitin ligase, whose depletion has been previously shown to inhibit S6 and 4E-BP phosphorylation while upregulating Akt(Ser473) phosphorylation. CUL4 promotes the degradation of REDD1 and TSC2, however these events are irrelevant to mTORC1 regulation in the TSC1 null MEFs. Recently, it has been shown that the DDB1-CUL4 ubiquitination of raptor is required for the stability of the mTOR complex1 [18]. It is unclear whether the loss of this function of DDB1 accounts for the ability of DDB1 RNAi to inhibit S6 phosphorylation, inasmuch as the RNAi pools against the other components of this ubiquitin ligase, CUL4A and RBX1/ROC1, did not inhibit S6 phosphorylation in MIA PaCa-2 cells (S1 Table).

### Other categories of S6-P regulators in TSC1 null MEFs

#### Mitochondrial Proteins

Among the 76 MEF2/3 positives are 6 mitochondrial proteins (Table 1). Among the mRNAs showing significantly reduced %S6-P in 1 of 3 MEF replicates were another four encoding mitochondrial proteins as well as the cytosolic proteins required for coenzyme synthesis, i.e. CoenzymeA Synthase/COASY, Pantothenic acid kinase-4/PANK4 and Thiamine phosphokinase/TPK1 (Table 2). CoenzmeA Synthase has been detected in a stable complex with S6KB1, but overexpression of COASY does not affect S6K activity and S6K does not phosphorylate COASY [59]. We have not determined whether depletion of these elements affects ATP content or activates AMPK. In addition, Duran et.al. [60] reported that the inhibition of mTORC1 signaling to S6K1 engendered by withdrawal of leucine could be rescued by provision of esterified versions of αKG. Depletion of Glutamate Dehydrogenase (GLUD1) did not inhibit S6 phosphorylation in Mia-Pa Ca 2 cells. Nevertheless, propionyl CoA carboxylase/PCCA and methylmalonyl CoA mutase, both of which were confirmed in 2/3 positives in the MEFs (Table 1) constitute an anaplerotic pathway that generates succinate from valine, isoleucine and odd chain fatty acids. Tumor cells utilize a variety of anaplerotic mechanisms to replace the TCA intermediates diverted to macromolecular synthesis [61] and those of most importance to MIA PaCa-2 cells remains to be determined.

#### DNA repair related hits

The considerable number of MEF2/3 positives concerned with DNA structure and repair (Tables 1,2) is unexpected and raises consideration regarding the relationship between DNA damage and mTORC1 activity. DNA damage uniformly inhibits mTORC1 by ATM/P53/TSC activation [62], although doxyrubicin-induced DNA damage is reported to activate mTORC1/S6K through p38α and TSC [63]. These responses however are presumably inoperative in the TSC1 null MEF screen. It is possible that the disordered genetic background of MiaPaCa cells and the TSC1 null MEFs creates a context in which DNA modification and/or repair is required to sustain S6 phosphorylation. However, the underlying mechanism and physiologic relevance of this phenomenon are unknown.

## Conclusion

A genome wide screen for S6 phosphorylation has uncovered a large number of gene products whose depletion reduces mTORC1 signaling. Previously identified regulators have been retrieved, including a number whose orthologues are also necessary for optimal TORC1 signaling in S. cerevisiae, as well as many genes not previously associated with mTORC1 regulation. Additional work will be required define the specific biochemical pathways that link these elements to mTOR complex 1. Imasmuch as many cancers exhibit mTOR hyperactivation, the identification of previously unappreciated proteins needed for maintenance of mTORC1 activity may provide new drug targets and lead to the development of beneficial therapies for tumors sensitive to mTOR inhibition. The 43 genes found to match the mTOR essentiality profile in the 216 different cancer cell lines represented in the Achilles dataset may help delineate a more general cellular state characterized by co-dependency with mTOR. This finding provides the rationale for selecting this set of genes for further analysis as candidate drug targets and for investigating synthetic lethal relationships with known mTOR inhibitors.

## Supporting Information

S1 Fig Identification of the PANTHER categories overrepresented among the confirmed “S6-P positives” from MiaPaCa cells. The 632 confirmed “S6-P positive” human genes were analyzed by PANTHER according to Biological Processes, Molecular Function and Protein Class and the subcategories that are enriched over their abundance in the genome are shown, ranked by the—log p value. A non-redundant list of the 217 specific genes comprising the categories shown under Biological Processes is provided in Supplemental S7 Table, and their distribution by Molecular Function is shown in Fig. 3D.
(TIF)

S2 Fig Classification by Molecular Function of the S6-P positives identified in TSC1-null mouse embryo fibroblasts. A. Functional categories of S6-P positives confirmed in 2 or more replicates, corresponding to Table 1. B. Functional categories of S6-P positives confirmed in one of three replicates, corresponding to Table 2. C. Functional categories of S6-P positives confirmed in any replicate, corresponding to the combined sets in Tables 1 and 2.
(TIF)

S1 Table Results of primary screen in MiaPaCa cells. Primary screen, arranged by plate. Avg.% S6P positive shown for each replicate plate. N = scramble RNAi (negative control); P = mTOR RNAi (positive control); X = experimental RNAi.
(XLSX)

S2 Table RNAi pool positives excluded because cell numbers were reduced >2S.D. below cell number seen with mTOR RNAi. Primary S6P positives not retested due to reduced cell number > 2SD below the mTOR RNAi on plates used for scoring. N = scramble RNAi (negative control); P = mTOR RNAi (positive control); X = experimental RNAi.
(XLSX)

S3 Table 1046 “primary S6P positives” from MiaPaCa cells. **Sheet A:** Primary positives chosen for retesting with 4 individual RNAi-see Supp Table 4; results shown here are from initial testing with RNAi pool **Sheet B: Primary S6P positives not retested with individual RNAis.** Primary S6P positives NOT selected for retesting with the 4 individual RNAi used in the intial screen.
(XLSX)

S4 Table Results of retesting 870 “primary S6P positives” with individual RNAis. Number of RNAi scoring positive.
(XLSX)

S5 Table All genes scored in primary screen ranked by avg. z or Q. Rank by avg. z of all genes, including only the plates used for scoring.
(XLSX)

S6 Table Performance of putatively known mTORC1/S6-P regulators in primary Screen. The effect of RNAi pools directed against putatively known regulators of S6-P in the MiaPaCa cell primary screen. Blue identifies plates not included in the scoring due to unacceptably low plate z’ values.
(XLSX)

S7 Table Genes comprising the PANTHER subcategories of “Biological Processes” that are overrepresented in comparison to the whole genome (See S1 Fig.). The nonredundant MiaPaCa genes contained in the overrepresented subcategories of Biological Processes (see S1 Fig.) classified by Molecular Function in Fig. 3D.
(XLSX)

S8 Table 534 genes tested with RNAi pools in TSC1 null Mouse Embryonic Fibroblasts. RNAi corresponding to 98/632 “confirmed S6P positives” identified in the primary screen were unavailable.
(XLSX)
