# Supplementary figures and images for: A Genome-Wide siRNA Screen in Mammalian Cells for Regulators of S6 Phosphorylation
Source: PLoS One. 2015 Mar 19;10(3):e0116096. doi: 10.1371/journal.pone.0116096 (PMC4366019; doi:10.1371/journal.pone.0116096)

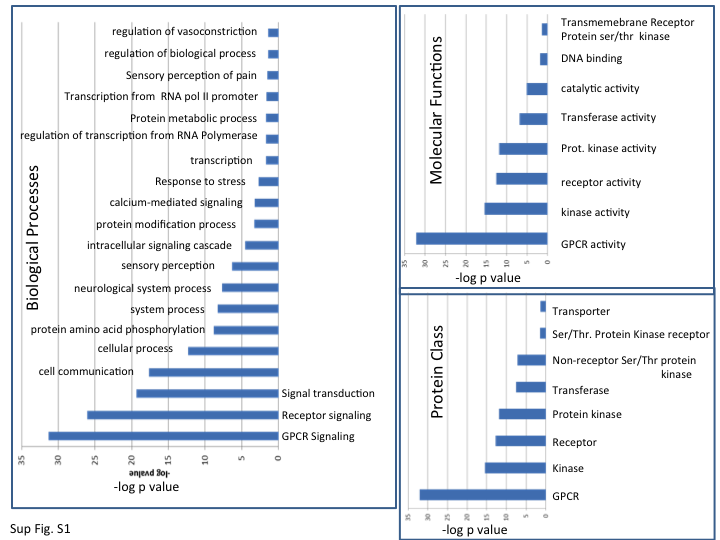

Supplement: S1 Fig — The 632 confirmed “S6-P positive” human genes were analyzed by PANTHER according to Biological Processes, Molecular Function and Protein Class and the subcategories that are enriched over their abundance in the genome are shown, ranked by the—log p value. A non-redundant list of the 217 specific genes comprising the categories shown under Biological Processes is provided in Supplemental S7 Table, and their distribution by Molecular Function is shown in Fig. 3D. (TIF) [file pone.0116096.s001.tif]

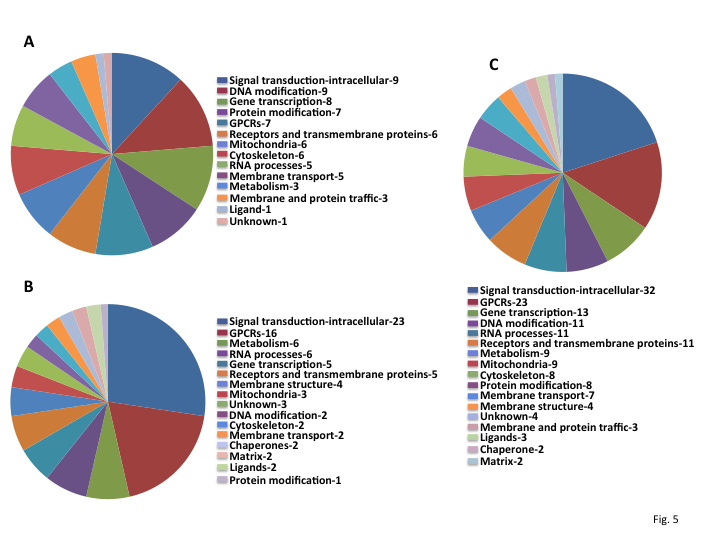

Supplement: S2 Fig — A. Functional categories of S6-P positives confirmed in 2 or more replicates, corresponding to Table 1. B. Functional categories of S6-P positives confirmed in one of three replicates, corresponding to Table 2. C. Functional categories of S6-P positives confirmed in any replicate, corresponding to the combined sets in Tables 1 and 2. (TIF) [file pone.0116096.s002.tif]
